# Perceptions, knowledge and attitudes towards the concept and approach of palliative care amongst caregivers: a cross-sectional survey in Karachi, Pakistan

**DOI:** 10.1186/s12904-020-00688-w

**Published:** 2020-11-26

**Authors:** Sameena Shah, Faizan Qaisar, Iqbal Azam, Khairunnisa Mansoor

**Affiliations:** 1 Department of Family Medicine, Campbell River Hospital, Vancouver Island Health Authority, Campbell River, Vancouver Island, BC Canada; 2 Department of Family Medicine, Aga Khan University and Hospital, Stadium Road, Karachi, Pakistan; 3 Ali Medicare, Karachi, Pakistan; 4 Aga Khan University and Hospital, Karachi, Pakistan; 5 Department of Community Health Sciences, Aga Khan University Medical College, Karachi, Pakistan; 6 School of Nursing and Midwifery, SONAM, Aga Khan University and Hospital, Karachi, Pakistan

**Keywords:** Palliative care, Caregivers, Comprehension, Attitudes, Terminal illness

## Abstract

**Background:**

Limited comprehension of the concept of palliative care and misconceptions about it are barriers to meaningful utilisation of palliative care programs. As caregivers play an integral role for patients with terminal illness, it is necessary to assess their perceptions and attitudes towards the palliative care approach.

**Method:**

A cross-sectional survey was conducted. Data was collected from the Aga Khan Hospital in-patient and out-patient departments and home-based palliative care services. All adult caregivers who met the inclusion criteria and consented, completed a questionnaire till the sample size was reached. Univariate and multivariate multivariable analysis was done and results were reported as crude prevalence’s, crude and adjusted prevalence ratios with 95% confidence intervals using Cox-proportional hazard algorithm. Mean difference of knowledge and attitude scores by caregiver variables were assessed using one-way ANOVA. SPSS version 18 was used and a *p*-value of less than 5% was treated as significant.

**Results:**

Out of 250 caregivers more than 60% were 40 years or less, majority were males and at least graduates. Approximately 70% of the respondents agreed with the statement that the person suffering from cancer should be informed about the diagnosis and disease progression. About 45% (95% C.I.: 39.03, 51.37%) of the study respondents had enhanced understanding about palliative care. Individuals under 40 years old, those with an education level of at least grade 10, children or relatives were found to have significantly more enhanced knowledge about palliative care. The majority believed that the patient should be informed about the diagnosis and should be facilitated to carry out routine activities and fulfill their wishes.

**Conclusion:**

Nearly half of the caregivers had enhanced understanding of the palliative care approach. They showed consistent understanding of two foundational aspects indicating correct knowledge across age groups, gender, education level, and relationship with the patient. Firstly, that palliative care should be offered to everyone suffering from a terminal illness and, secondly, that this approach encompasses not just physical, but also psychological and social needs of the patient and the family. These findings will help inform the establishment of a palliative care program that fills the gaps in comprehension and knowledge of caregivers.

## Background

The World Health Organization (WHO) defines palliative care (PC) as “an approach that improves the quality of life of patients and their families facing the problems associated with life-threatening illness, through the prevention and relief of suffering by means of early identification and impeccable assessment and treatment of pain and other problems, physical, psychosocial and spiritual.” [1]. Furthermore, this approach extends to include the family members not just during the life, but also after the death of the patient. The PC approach, given its holistic nature, is multi-disciplinary and is based on a team that includes physicians, nurses, social workers and many other allied health care workers. In addition, family members often take on an increasingly integral role as caregivers as disease progresses.

For purposes of clarity, the caregiver/s, are defined as a person/s who gives help and protection to someone such as a child, an old person, or someone who is sick [2]. The primary caregiver is usually the family member who spends the most time with the patient and is involved in the day to day care of the patient. However, there may be other paid caregivers who would also be in communication with physicians and nurses involved in patient care. In the context of PC in Pakistan - as in the rest of the world - this would be any individual who takes care of the patient, such as feeding, dressing or cleaning and making relevant day to day decisions. This person may also be involved in discussing patient condition or disease progress with the medical team or distant family members. In the Pakistani socio-cultural system, this is usually one or more close family member/s such as spouse, children, and/or siblings. However, occasionally, this individual may be a trained attendant, nurse or nursing assistant. The team addresses and prioritises the patient’s needs pertaining to physical, emotional, social, and spiritual dimensions of PC on a continuum while coordinating this care with all concerned [3].

By 2060, 83% of deaths worldwide due to health-related suffering will occur in low-income and middle-income countries which constitute about 47% of the world’s population [4]. Cancer is the second leading cause of deaths globally, 70% of which occur in low and middle income countries like Pakistan [4]. An ongoing study that is monitoring the development of palliative care services globally with the aim to categorize countries to levels of palliative care development, placed Pakistan in category 3a (Isolated palliative care provision) in 2017 [5]. It also notes that 47.5% of the world’s population belongs to countries that fall within this category [5]. In Pakistan, there are only a handful of medical institutes that offer palliative care [6]. Given these circumstances, there is a great need to develop and integrate palliative care into health systems at a public health level.

Among several challenges to developing a public health approach, one is the paucity of public awareness of palliative care, as demonstrated by numerous international surveys [7–10]. Moreover, the understanding of the situation is complicated by limited research in this relatively new field in Pakistan [6, 11, 12]. Lack of comprehension of the concept and scope of PC and misconceptions about it are a barrier to the uptake and meaningful utilisation of this comprehensive approach by patients and families [5, 11–13]. Misperceptions are common in the public and amongst caregivers and can adversely impact the uptake of this beneficial service even when it is available [10]. This compounds the stress and burden of all caregivers looking after terminally ill patients. To ensure meaningful and successful uptake, potential caregivers, whether family members or professionals, need to have knowledge and understanding of the benefits of this approach in the care of their patients [14–18]. The objectives of this study, therefore, were to determine knowledge, in terms of perceptions and attitudes of caregivers, about the concept and principles of palliative care. The findings will help to inform interventions to enhance knowledge and address misperceptions, as palliative care becomes more widely available in Pakistan.

## Methods

A cross-sectional survey was conducted over a period of one year on (*n* = 250) primary caregivers to assess their perceptions, attitudes and knowledge about PC after obtaining approval from the Ethics Research Committee of the Aga Khan University Hospital (AKUH). Data was collected from the Aga Khan Hospital in-patient and out-patient departments and home-based palliative care services from mid 2015 to 2016. The Aga Khan University Hospital is a tertiary care hospital with outreach clinics and a home-based care service in the metropolitan city of Karachi.

The sample size estimate was based on knowledge of PC and anticipated as 50% with 5% level of significance, 6.5% precision and 10% incompleteness or refusal to participate. The study participants were recruited from the in-patient cancer, neurology, pulmonary and cardiac wards, outpatient consultant clinics, and patients’ homes during home visits. The inclusion criteria were any person (female or male) over the age of 18 years who was taking care of a patient suffering from a terminal illness, was aware of the diagnosis and the prognosis, who conformed to the operational definition of caregiver, and who gave informed consent. The three exclusion criteria were those under 18 years of age, those who refused consent, and/or those who did not conform to the operational definition of primary caregivers. A questionnaire was developed in both Urdu and English which was cross validated and piloted on a sample drawn from the same study population, meeting the same inclusion criteria, before being used for data collection.

To ensure face and content validity, the team providing palliative care reviewed papers describing similar surveys [19, 20] assessing knowledge, believes and attitudes about PC. This was followed by a further review by the palliative care team to ensure that the questions covered aspects of palliative care comprehensively. The questions were further screened to make sure that they were relevant to the Pakistani context and setting. Some of the questions were generic in terms of the meaning and principles of PC whereas others were regarding attitudes towards core concepts of PC. A section covering demographic and social aspects such as, gender, educational qualifications, and relationship with the patient was included. The questionnaire was then reviewed by the statistician. The questionnaire was first developed in English and then translated into Urdu and back-translated into English by different people for cross validation. The questionnaire was piloted on the same population before data collection. A few modifications were made after it was piloted. All study participants were approached by the nursing staff or the home-based family physician in the selected study sites. All those who met the inclusion criteria were asked to complete the questionnaire till the sample size was reached. All participants were able to read and write in at least one of the two languages. To ensure confidentiality, the names of the participants were replaced by numbers throughout analysis. The original hard copy questionnaires were stored in a locked filing cabinet with only the principle investigator having access to them.

### Data analysis

The data was double entered by two data entry operators in Epidata, then verified for data entry errors and cleaned. The cleaned data was then converted into SPSS for analysis. Frequency distributions of caregiver age, gender, level of education, relationship with the patient, attitudes about whether the person receiving palliative care should be allowed to carry out normal routine activities or to fulfill all his/her wishes and whether a person should be given a diagnosis of cancer or disease progression, were generated.

Several outcomes were assessed. One was binary for which prevalence ratios with 95% CI have been reported. Prevalence with 95% confidence interval for knowledge of palliative care was calculated. Scores were derived for correct answers of the knowledge component and compared with demographic variables. The association of knowledge of palliative care with caretakers’ age, gender, level of education and relationship with the patient was assessed using univariate and multivariable analysis and results were reported as crude prevalence’s, crude and adjusted prevalence ratios with 95% confidence intervals using Cox-proportional hazard algorithm. Each question related to perceptions and attitudes was given a score of 1 when positive, otherwise a score of zero was given. These scores were then aggregated to obtain final correct knowledge and attitude scores for different aspects of PC. Scores for the following knowledge variables were calculated: goals of PC, focus of PC, composition of PC team, setting of PC, knowledge about hospice, perceptions about provider of PC, perceptions about needs of patients suffering from a terminal illness, perceptions about whether PC should be offered to people diagnosed with a terminal illness, perceptions about the best setting to provide PC and perceptions about the best source for obtaining knowledge about PC.

Mean difference of these scores by primary caregivers age group, gender, education level and relationship with the patient was assessed using one-way ANOVA. SPSS version 18 was used to analyze the data. A *p*-value of less than 5% was treated as significant.

## Results

A total of (*n* = 250) primary caregivers completed the self-administered questionnaire. More than 60% of them were 40 years old or less and the majority were males. More than 85% of them were at least graduates. More than 50% of the caregivers were close family members, such as a spouse or a child. About 45% (95% C.I.: 39.03, 51.37%) of the study respondents had a reasonable amount of knowledge about palliative care. The caregivers who were found to have significantly better comprehension about palliative care were young individuals aged < 40 years, those with an education level of up to grade 10, and/or children of the patient. (Table 1) More than half of them agreed that the person receiving palliative care should be encouraged to carry out normal routine activities or to fulfill their wishes. Approximately 70% of the respondents agreed with the statement that the person suffering from cancer should be informed about the diagnosis and disease progression. (Table 2) A little less than half 113 (45.2%) agreed with the statement describing palliative care in the questionnaire, while 103 (41.2%) did not know and 34 (13.6%) disagreed with the statement. The majority agreed with the statement about the meaning of palliative care.

**Table 1:** Association of Different Socio-Demographic Distribution of Respondents with Correct Comprehension of the Concept and Principles of Palliative Care (Crude and Adjusted Prevalence Ratios with 95%

		Prevalence of Palliative Care Knowledge (***n*** = 113/250)	Crude Prevalence Ratio (95% CI)	Adjusted Prevalence Ratio (95% CI)
**Age Group (in years)**	< 30 (Ref.)	58.7%	1	1
	30–40	62.3%	1.06 (0.70, 1.61)	0.74 (0.42, 1.28)
	40–50	12.9%	0.22 (0.11, 0.44)	0.23 (0.11, 0.48)
	50–60	44.4%	0.76 (0.40, 1.42)	0.66 (0.34, 1.31)
**Gender**	Male	46.4%	1.07 (0.73, 1.56)	
	Female (Ref.)	43.4%	1	
**Qualification**	Up to Secondary	85.7%	1.79 (1.13, 2.83)	1.96 (1.09, 3.51)
	Graduate	31.1%	0.65 (0.42, 1.00)	0.49 (0.29, 0.84)
	Masters (Ref.)	47.9%	1	1
**Relationship with patient**	Self/Spouse (Ref.)	21.43%	1	1
	Children	46.48%	2.17 (1.04, 4.53)	1.01 (0.44, 2.34)
	Others	51.82%	2.42 (1.21, 4.84)	2.73 (1.25, 5.97)

**Table 2:** Frequency of Level of Agreement or Disagreement of Respondents with Core Concepts of Palliative Care (*n* = 250)

CORE CONCEPT	RESPONSE	***N*** (%)
Person receiving palliative care should be allowed to carry out normal routine activities	Strongly Agree	76 (30.4%)
	Agree	76 (30.4%)
	Disagree	10 (4.0%)
	Strongly Disagree	19 (7.6%)
	I Don’t Know	69 (27.6%)
	**Total**	**250**
Person receiving palliative care should be allowed to fulfill all his/her wishes	Strongly Agree	130 (52.0%)
	Agree	43 (17.2%)
	Disagree	11 (4.4%)
	Strongly Disagree	17 (6.8%)
	I Don’t Know	49 (19.6%)
	**Total**	**250**
Person suffering from cancer should be given diagnosis or disease progress information if s/he wants to know.	Strongly Agree	57 (22.8%)
	Agree	102 (40.8%)
	Disagree	24 (9.6%)
	Strongly Disagree	19 (7.6%)
	I Don’t Know	48 (19.2%)
	**Total**	**250**

Differences in the mean scores of knowledge of different aspects of palliative care by age, gender, level of education and relationship with the patient were observed. (Table 3).

**Table 3:** Score and Mean (SD) of Perceptions and Knowledge about Different Aspects of Palliative Care by Socio-Demographic Characteristics

Characteristics.	***n***	Goals of PC Mean (SD)	Focus of PC Mean (SD)	Team Members of PC Mean (SD)	Settings of PC Mean (SD)	Knowledge about Hospice Mean (SD)	Provider of PC Mean (SD)	Fulfilling Needs of Patients Suffering from a Terminal Illness Mean (SD)	Palliative Care Should be Offered to Everyone Suffering from a Terminal Illness Mean (SD)	Best Setting for Providing PC Mean (SD)	Best Source of Obtaining Knowledge About PC Mean (SD)
**Overall**	**250**	**1.37 (0.97)**	**2.21 (1.42)**	**2.23 (1.38)**	**1.95 (1.14)**	**1.17 (1.06)**	**1.34 (1.78)**	**1.41 (0.75)**	**4.62 (4.15)**	**1.47 (1.09)**	**2.41 (1.83)**
***p*-value Age group**		< 0.001	< 0.001	< 0.001	< 0.001	< 0.001	< 0.001	< 0.873	< 0.001	< 0.001	< 0.003
**Age Group**											
< 30	92	1.80 (0.76)	2.33 (1.08)	2.51 (0.90)	2.15 (1.33)	1.32 (1.33)	1.85 (1.92)	1.41 (0.84)	4.53 (3.48)	1.75 (1.22)	2.59 (2.30)
30–40	61	1.34 (0.96)	2.00 (1.43)	1.07 (1.17)	1.10 (1.04)	0.75 (0.77)	0.26 (0.73)	1.44 (0.67)	1.49 (2.44)	0.98 (1.02)	1.75 (1.49)
40–50	70	0.93 (1.00)	1.66 (1.59)	2.97 (1.64)	2.29 (0.57)	1.23 (0.94)	1.29 (1.96)	1.36 (0.76)	5.71 (4.65)	1.20 (0.79)	2.44 (1.30)
50–60	27	1.11 (1.01)	3.74 (0.71)	2.00 (0.28)	2.30 (0.82)	1.44 (0.58)	2.15 (1.29)	1.48 (0.58)	9.15 (1.98)	2.30 (0.67)	3.22 (1.4
***p*-value Gender**		< 0.279	< 0.001	< 0.061	< 0.009	< 0.025	< 0.001	< 0.001	< 0.195	< 0.049	< 0.001
**Gender**											
Male	151	1.32 (0.99)	2.50 (1.43)	2.36 (1.41)	1.79 (1.04)	1.05 (0.86)	0.73 (1.26)	1.22 (0.69)	4.34 (4.39)	1.36 (0.97)	2.11 (1.73)
Female	99	1.45 (0.95)	1.77 (1.3)	2.03 (1.3)	2.18(1.26)	1.35 (1.28)	2.26 (2.04)	1.71 (0.74)	5.04 (3.74)	1.64 (1.25)	2.88 (1.89)
***p*-value Education**		<.0.001	< 0.001	< 0.001	< 0.136	< 0.001	< 0.001	< 0.001	< 0.001	< 0.001	< 0.017
**Education**											
Upto Secondary	35	1.971(.38)	2.66(.99)	2.08(.44)	1.97(.38)	0.94(.48)	3.05 (2.04)	1.89 (0.323)	7.05 (2.04)	2.25(.61)	2.14 (1.22)
Inter. to Graduate	119	1.21 (1.19)	1.32 (1.36)	2.57 (1.75)	2.08 (1.18)	1.51 (1.22)	1.100 (1.66)	1.61 (0.71)	5.30 (4.37)	1.33 (1.19)	2.75 (1.8)
Postgraduate & above	96	1.35 (1.37)	3.12 (2.21)	1.85 (2.23)	1.77 (1.95)	0.82 (1.17)	1.00 (1.34)	0.99 (0.703)	2.88 (4.62)	1.34 (1.47)	2.08 (2.41)
***p*-value Relationship**		< 0.001	< 0.001	< 0.776	< 0.001	< 0.001	< 0.001	< 0.051	< 0.207	< 0.001	< 0.042
**Relationship**											
Self/Spouse	42	0.90 (0.79)	2.93 (1.04)	2.12 (0.88)	2.83 (0.69)	0.81 (0.94)	1.62 (1.36)	1.17 (0.66)	4.48 (4.44)	1.88 (0.80)	3.00 (1.63)
Children	71	1.62 (0.62)	2.76 (1.20)	2.31 (0.95)	1.72 (1.13)	0.69 (0.60)	2.03 (1.97)	1.41 (0.96)	5.35 (3.83)	1.89 (1.33)	2.48 (2.23)
Others	137	1.39 (1.12)	1.71 (1.43)	2.23 (1.66)	1.80 (1.14)	1.53 (1.14)	0.89 (1.65)	1.49 (0.63)	4.28 (4.21)	1.12 (0.93)	2.20 (1.62)

### Age

Goals of PC Score (F = 13.23; df = 3246; *p*-value< 0.001), Focus of PC Score (F = 17.50; df = 3246; *p*-value< 0.001), Team Members of PC Score (F = 31.08; df = 3246; *p*-value< 0.001), Settings of PC Score (F = 18.15; df = 3246; *p*-value< 0.001), Hospice Knowledge Score (F = 4.58; df = 3246; *p*-value< 0.001), Perceptions about Provider of PC Score (F = 13.65; df = 3246; *p*-value< 0.001), Perceptions that Palliative Care should be Offered to Everyone Suffering from a Terminal Illness Score (F = 33.16; df = 3246; *p*-value< 0.001), Perceptions about Best Setting to Provide PC Score (F = 14.64; df = 3246; *p*-value< 0.001) and Perceptions about the Best Source of Knowledge about PC Score (F = 4.89; df = 3246; *p*-value = 0.003) were found to be significantly different by the respondents’ age except Perceptions about the Needs of Patients Suffering from a Terminal Illness Score (F = 0.233; df = 3246; *p*-value = 0.873). Focus of PC Score (F = 17.02; df = 1248; *p*-value< 0.001).

### Gender

Settings of PC Score (F = 7.002; df = 1248; p-value = 0.009), Knowledge of Hospice Score (F = 5.12; df = 1248; *p*-value = 0.025), Perceptions about Provider of PC Score (F = 53.94; df = 1248; *p*-value< 0.001), Perceptions about the Needs of Patients Suffering from a Terminal Illness Score (F = 28.02; df = 1248; *p*-value< 0.001), Perceptions of Best Setting to provide PC Score (F = 3.93; df = 1248; *p*-value = 0.049) and Perceptions about the Best Source of Knowledge about PC Score (F = 11.09; df = 1248; *p*-value = 0.001) were found significantly different by gender of the respondent except Goals of PC Score (F = 1.177; df = 1248; *p*-value = 0.279), Team Members of PC Score (F = 3.55; df = 1248; *p*-value = 0.061) and Perceptions that Palliative Care should be Offered to Everyone Suffering from a Terminal Illness Score (F = 1.69; df = 1248; *p*-value = 0.195).

### Education

Goals of PC Score (F = 8.80; df = 2247; p-value< 0.001), Focus of PC Score (F = 73.13; df = 2247; p-value< 0.001), Team Members of PC Score (F = 8.03; df = 2247; *p*-value< 0.001), Hospice Knowledge Score (F = 13.42; df = 2247; *p*-value< 0.001), Perceptions about Provider of PC Score (F = 22.43; df = 2247; *p*-value< 0.001), Perceptions about the Needs of Patients Suffering from a Terminal Illness Score (F = 33.24; df = 2247; *p*-value< 0.001), Perceptions that Palliative Care should be Offered to Everyone Suffering from a Terminal Illness Score (F = 18.25; df = 2247; *p*-value< 0.001), Perceptions of Patient Best Setting to Provide PC Score (F = 11.47; df = 2247; *p*-value< 0.001) and Perceptions about the Best Source of Knowledge about PC Score (F = 4.13; df = 2247; *p*-value = 0.017) were found significantly different by level of education except Settings of PC Score (F = 2.01; df = 2247; *p*-value = 0.136).

### Relationship with patient

Goals of PC Score (F = 7.25; df = 2247; *p*-value = 0.001), Focus of PC Score (F = 22.53; df = 2247; p-value< 0.001), Settings of PC Score (F = 17.18; df = 2247; *p*-value< 0.001), Hospice Knowledge Score (F = 20.15; df = 2247; p-value< 0.001), Perceptions about Provider of PC Score (F = 11.03; df = 2247; *p*-value< 0.001) and Perceptions about Best Setting to Provide PC Score (F = 16.87; df = 2247; *p*-value< 0.001), Perceptions about the Best Source of Knowledge about PC Score (F = 3.21; df = 2247; *p*-value = 0.042) were found significantly different for relationship with patient except Team Members of PC Score (F = 0.25; df = 2247; p-value = 0.776), Perceptions about the Needs of Patients Suffering from a Terminal Illness Score (F = 3.01; df = 2247; p-value = 0.051) and Perceptions that Palliative Care should be Offered to Everyone Suffering from a Terminal Illness Score (F = 1.58; df = 2247; *p*-value = 0.207) (Table 3).

## Discussion

The concept of PC as a formal approach is relatively new to Pakistan, within the community as well as amongst its physicians. Just a handful of studies have been done on this concept in Pakistan [6, 11, 12]. Aga Khan University Hospital set up a formal palliative care program in 2012 consisting of a section of palliative medicine in the department of oncology, with dedicated clinics and home-based care services. Simultaneously, an online course and workshops on PC were developed for health care professionals [21]. However, awareness in the community at large appears to be limited as it remains a new concept in Pakistan. All the respondents in this study were caring for patients with terminal illnesses and were, therefore, able to assess the needs of these patients in terms of symptom relief. They were also aware of the care and treatment being provided for the patient whether in hospital, at home or in the outpatient clinics. The vast majority, 223 (89%) out of the total 250 study participants, were young adults up to 50 years old, whereas the remaining few were 50 to 60 years old. This is in keeping with the fact that caregivers are more likely to be younger and able-bodied, as caring for palliative care patients can be increasingly demanding as the patient’s condition progresses. More than half of the caregivers were male which is in contrast with international studies that have repeatedly found that the majority of caregivers are female [22].

This can be explained by the patriarchal structure of Pakistani culture including its households [23]. The survey was done on patients accessing a relatively expensive private pay hospital in Karachi, which has a literacy rate of over 75% [24]. However, the fact that all of the respondents were literate, in a country where the adult literacy rate (over 15 years) was 59% in 2017 and the majority of the study participants were at least graduates, may have had a bearing on the levels of awareness and knowledge about PC [25]. A study in the US found a similar association [18].

In this sample, half the caregivers were close relatives of the patient, such as wife, husband, sister or brother in almost equal numbers, while the remaining were nurses, nursing attendants or distant relatives. This matches findings in international studies where close family members take on the role of caregiver when a spouse, parent, child or relative falls ill [26, 27] (Table 1).

Just over half (54.8%) did not know or did not comprehensively understand the concept of PC while less than half of the caregivers had enhanced understanding of the basic concept of PC. It is noteworthy that these numbers are comparable to a large study done in the US in 2019, because the same percentage of caregivers (55%), had never heard of PC, whereas 19.2% knew what PC was and believed they would be able to explain it to someone else [18, 28]. A study done on the general population in the US, found misperceptions and negative attitudes towards palliative care even though palliative care is more widely available there [29].

The PC approach works in tandem with the ‘patient centered approach’ that is based on the principle that the patient should be given every opportunity to be involved in all aspects of care starting from complete information about the terminal illness to decision making about treatment options, unless the patient forgoes this right or defers it to a family member [30–32]. These principles underpin the ethical and professional obligations of this approach [33]. In keeping with this, the majority of the respondents believed that the patient should be informed about the diagnosis of a terminal illness, which matches the findings reported in an earlier paper done in the community health center of the same hospital and international studies done in hospitals and the community [30, 32, 33]. This finding should give more confidence to physicians to ask the patient if s/he wants to know the diagnosis and discuss management options. This is especially relevant when relatives insist that it should not be disclosed or discussed with the patient, fulfilling the ethical principle of patient autonomy [30–32]. Similarly, the majority of the respondents believed that patients should be encouraged to carry out routine activities and be facilitated to fulfill their wishes. This is in accordance with the PC approach wherein patients are enabled to live normal lives and every effort is made to fulfill their wishes to prevent helplessness and suffering [1, 19] (Table 2).

In contrast to a focused medical approach, the goals of palliative care, therefore, are to ease the suffering of the patient and the family across all its domains, while remembering to reassess at every step to ensure that futile treatments and false hope are avoided [1]. While PC can be provided in hospital, it can also be provided at home, in clinics and hospice (a place where people with terminal illnesses can pass their last months of life) [34]. Younger caregivers were aware of this, probably because this group was more likely to have greater exposure to multiple sources of information, even if they had been less likely to have had any personal experience of caring for a relative suffering from a terminal illness [19, 29]. Similarly, the oldest age group also had an enhanced understanding and knowledge of the concept and principles of PC.

The obvious explanation is their greater experience and therefore first-hand learning regarding this aspect of life, because by this stage in their lives they would have been more likely to have observed and/or been involved in the care of a family member or a friend suffering from a terminal illness. All age groups believed that an essential aspect of PC was fulfilling the needs of patients suffering from a terminal illness (Table 3).

Participants educated up to grade 10 at the time of data collection were younger and had greater knowledge and correct perceptions of PC, whereas respondents with a higher level of education were older and had a limited understanding of the concept. Better knowledge and awareness in younger individuals has also been noted in a study from the US [29]. That there was less variation in accurate perceptions about these aspects of PC across gender is understandable as both have equal opportunity to care for a palliative patient, regardless of age and relationship with patient. However, males had a better comprehension of the focus of PC whereas more females had enhanced knowledge about where PC can be provided.

Children had significantly more information about PC compared to a spouse, again comparable internationally [29]. This is likely because younger caregivers have increased access to information technology, along with the education and skills to use it, relative to most patients’ spouses. This is possibly because, although affected emotionally, children take on more responsibility in the practical and medical aspects of caring for their parents as patients, are more involved in discussions and management aspects with the medical team, and therefore actively seek information to enhance and improve the care of their affected parent, sparing the other parent of this difficult task. This can also be considered reflective of traditional Pakistani cultural practices and the dearth of nursing homes and hospices such that children often take on the responsibility of caring for parents as they age and/or when they are sick [35–37] Table 3.

## Limitations

As the study setting has a gradually expanding PC program, the caregivers in the study sample may have acquired some information about the concept of PC during their interactions with the nurses and physicians. The results, therefore, should be generalized with caution to caregivers providing PC in settings without formal programs. This study was done on caregivers associated with a single hospital with a nascent palliative care program in a big city and the results are not generalizable to the rest of the country. All the respondents were literate, with majority of the caregivers educated at least up till grade 10, and so the perceptions, knowledge and attitudes of caregivers with less schooling than that - which in the Pakistani context is a sizeable majority - is not known [27]. Most of the respondents in the sample were males and may not be representative of the actual gender distribution in other samples and international populations where studies have shown that most caregivers are female. Additionally, this does not necessarily imply that most caregivers in this setting are male, so it may also not be representative of the actual gender distribution in this population of caregivers. Further studies assessing this aspect will make it clear.

## Conclusion

Nearly half of the caregivers had partial understanding of the holistic PC approach in terms of perceptions in this limited study. The respondents showed consistent understanding of two foundational aspects indicating correct knowledge across age groups, gender, education level, and relationship with the patient. Firstly, that PC should be offered to everyone suffering from terminal illness [20] and, secondly, that this approach encompasses not just physical, but also psychological and social needs. In Pakistan PC is not available in the public health system unlike in developed countries. Education and training about PC are not part of medical education in the large majority of medical institutions in the country. It has been established by the WHO and noted repeatedly in the literature that knowledge deficits and misunderstandings are amongst the main challenges in providing PC [37–39]. Thus, it is necessary to assess perceptions and knowledge of PC in caregivers on a wider scale as palliative care programs are established, while simultaneously incorporating training in PC in the health care professions within a public health system [36, 37, 39].

This study’s findings will enable the development of focused education strategies to fill the knowledge gaps in the Pakistani context. Even when health care providers or physicians are reluctant to offer PC as an option due to feelings of failure or lack of information, they will be more likely to offer PC if caregivers or the patient are knowledgeable about it and request it [13, 14]. It is essential to increase awareness and enhance knowledge about PC in both patients and caregivers while simultaneously dispelling misperceptions as PC services are developed at a public health level [14, 32, 38, 39].

## Data Availability

All the data and material used in the conduct of this study is available for review from the Authors.

## Abbreviations

WHO: World Health Organization
PC: Palliative Care
AKUH: Aga Khan University and Hospital

## References

[1] World Health Organization. WHO Definition of Palliative Care [accessed on December, 2019]. Available from: https://www.who.int/cancer/palliative/definition/en/.

[2] Merriam-Webster. Caregiver [accessed on January, 2019]. Available from: https://www.merriam-webster.com/dictionary/caregiver.

[3] Higginson IJ, Evan CJ (2010). What is the evidence that palliative care teams improve outcomes for cancer patients and their families?. Cancer J Sci Am.

[4] Sleeman KE, de Brito M, Etkind S, Nkhoma K, Guo P, Higginson IJ, et al. The escalating global burden of serious health-related suffering: projections to 2060 by world regions, age groups, and health conditions. Lancet Glob Health. 2019;7(7):883-892. Available from: doi:10.1016/S2214-109X(19)30172-X. Epub. 10.1016/S2214-109X(19)30172-X PMC6560023 31129125

[5] Clark D, Baur N, Clelland D, Garralda E, López-Fidalgo J, Connor S, et al. Mapping levels of palliative care development in 198 countries: the situation in 2017. J Pain Symptom Manag. 2019;59(4):749–807.e4 Available from: doi: 10.1016/j.jpainsymman.2019.11.009. Epub. 10.1016/j.jpainsymman.2019.11.009 PMC7105817 31760142

[6] Shad A, Ashraf MS, Hafeez H (2011). Development of palliative-care services in a developing country: Pakistan. J Pediatr Hematol Oncol.

[7] Docherty A, Owens A, Asadi-Lari M, Petchey R, Williams J, Carter YH (2008). Knowledge and information needs of informal caregivers in palliative care: a qualitative systematic review. Palliat Med.

[8] Benini F, Fabris M, Pace DS, Verno V, Negro V, de Conno F (2011). Awareness, understanding and attitudes of Italians regarding palliative care. Ann Ist Super Sanita.

[9] Matsuyama RK, Balliet W, Ingram K, Lyckholm LJ, Wilson-Genderson M, Smith TJ (2011). Will patients want hospice or palliative care if they do not know what it is?. J of Hosp Palliat Nurs.

[10] Hirai K, Kudo T, Akiyama M, Matoba M, Shiozaki M, Yamaki M (2011). Public awareness, knowledge of availability, and readiness for cancer palliative care services: a population-based survey across four regions in Japan. J Palliat Med.

[11] Abbas SQ, Muhammad SR, Mubeen SM, Abbas SZ (2004). Awareness of palliative medicine among Pakistani doctors: a survey. J Pak Med Assoc.

[12] Singh T, Harding R (2015). Palliative care in South Asia: a systematic review of the evidence for care models, interventions, and outcomes. BMC Res Notes.

[13] Love AW, Liversage LM (2014). Barriers to accessing palliative care: a review of the literature. Prog Palliat Care.

[14] McAteer R, Wellbery C (2013). Palliative care: benefits, barriers, and best practices. Am Fam Physician.

[15] Kavalieratos D, Corbelli J, Zhang D, Dionne-Odom JN, Ernecoff NC, Hanmer J (2016). Association between palliative care and patient and caregiver outcomes: a systematic review and meta-analysis. JAMA..

[16] Choi S, Seo J (2019). Analysis of caregiver burden in palliative care: an integrated review. Nurs Forum.

[17] Veloso VI, Tripodoro VA (2016). Caregivers burden in palliative care patients: a problem to tackle. Curr Opin Support Palliat Care.

[18] Dionne-Odom JN, Ornstein KA, Kent EE (2019). What do family caregivers know about palliative care? Results from a national survey. Palliat Support Care.

[19] Mcllfatrick S, Hasson F, McLaughlin D, Johnston G, Roulston A, Rutherford L (2013). Public awareness and attitudes toward palliative care in Northern Ireland. BMC Palliat Care.

[20] Wallace J. Public awareness of palliative care: report of the findings of the first national survey in Scotland into public knowledge and understanding of palliative care. Edinburgh (SCT): Scottish Partnership for Palliative Care; 2003. 22 p.

[21] The Aga Khan University. Networks of quality, teaching and learning [accessed on July, 2020]. Available from: https://www.aku.edu/events/pages/event-detail.aspx?EventID=460.

[22] Dahlberg L, Demack S, Bambra C (2007). Age and gender of informal carers: a population-based study in the UK. Health Soc Care Commun.

[23] Chauhan K. Patriarchal Pakistan: Women’s representation, gender inequality in the public sector in Pakistan. New York: Palgrave Macmillan; 2014. 57 p.

[24] Rehman A, Jingdong L, Hussain I (2015). The province-wise literacy rate in Pakistan and its impact on the economy. Pac Sci Rev B.

[25] The World Bank. Literacy rate, adult total (% of people ages 15 and above) [accessed on June, 2020]. Available from: https://data.worldbank.org/indicator/SE.ADT.LITR.ZS.

[26] Morris SM, King C, Turner M, Payne S (2015). Family carers providing support to a person dying in the home setting: a narrative literature review. Palliat Med.

[27] Ramirez A, Addington-Hall J, Richards M (1998). ABC of palliative care. The carers. BMJ.

[28] Bill McInturff and Elizabeth Harrington. Public Opinion Research on Palliative Care: A Report Based on Research by Public Opinion Strategies. New York (USA): Center to Advance Palliative Care;2003.3p.

[29] Taber JM, Ellis EM, Reblin M, Ellington L, Ferrer RA (2019). Knowledge of and beliefs about palliative care in a nationally-representative US sample. PloS One.

[30] Walczak A, Butow PN, Clayton JM, Tattersall MH, Davidson PM, Young J (2014). Discussing prognosis and end-of-life care in the final year of life: a randomised controlled trial of a nurse-led communication support programme for patients and caregivers. BMJ Open.

[31] Zafar W, Hafeez H, Jamshed A, Shah MA, Quader A, Yusuf MA (2016). Preferences regarding disclosure of prognosis and end-of- life care: a survey of cancer patients with advanced disease in a lower-middle-income country. Palliat Med.

[32] Ishaque S, Saleem T, Khawaja FB, Qidwai W (2010). Breaking bad news: exploring patient's perspective and expectations. J Pak Med Assoc..

[33] Mohanti BK (2009). Ethics in palliative care. Indian J Palliat Care.

[34] Merriam-Webster. Hospice [accessed on June, 2019]. Available from: https://www.merriam-webster.com/dictionary/hospice.

[35] Shad A, Ashraf MS, Hafeez H (2011). Development of palliative-care services in a developing country: Pakistan. J Pediatr Hematol Oncol.

[36] Aljawi DM, Harford JB. Palliative Care in the Muslim-Majority Countries: The Need for More and Better Care. In: Chang E, editor. Contemporary and Innovative Practice in Palliative Care. [Internet]. Shanghai:InTech; 2012.[2020 Jul 3]. Chapter 9. Available from: InTech-Palliative_care_in_the_muslim_majority_countries_the_need-for-more-and-better_care.pdf.

[37] Ddungu H (2011). Palliative care: what approaches are suitable in developing countries?. Br J Haematol.

[38] Maddocks I (2004). Palliative care education in the developing countries. J Pain Palliat Care Pharmacother.

[39] Stjernswärd J (2007). Palliative care: the public health strategy. J Public Health Policy.

